# Maltodextrin-Coated Peppermint and Caraway Essential Oils Effects on Soil Microbiota

**DOI:** 10.3390/plants11233343

**Published:** 2022-12-02

**Authors:** Maria Chmiel, Gabriela Drzymała, Jan Bocianowski, Andreja Komnenić, Agnieszka Baran, Agnieszka Synowiec

**Affiliations:** 1 Department of Microbiology and Biomonitoring, The University of Agriculture in Kraków, 30-059 Krakow, Poland; 2 Department of Mathematical and Statistical Methods, Poznań University of Life Sciences, 60-637 Poznań, Poland; 3 Department of Field and Vegetable Crops, Biotechnical Faculty Podgorica, University of Montenegro, 81000 Podgorica, Montenegro; 4 Department of Agricultural and Environmental Chemistry, The University of Agriculture in Krakow, 31-120 Krakow, Poland; 5 Department of Agroecology and Crop Production, The University of Agriculture in Krakow, 31-120 Krakow, Poland

**Keywords:** inhibitory effect, the serial dilution method, bacteria, actinomycetes, fungi

## Abstract

Essential oils exhibit strong antimicrobial effects that can serve as a substitute for synthetic pesticides. However, many reports mention the use of essential oils in protecting above-ground plant organs and storing raw materials and seeds, but only a few address the effects of treatments on soil microbiota. Regarding this, it is necessary to find a solution that will prevent the rapid degradation of oils in soil and extend the period of their action on the soil microbiota. The solution to this problem can be microencapsulation, where the choice of carrier plays a key role. In our experiment, maltodextrin was studied, often used in the microencapsulation of essential oils. It was examined independently in two doses (M1 and M2, with 50 and 200 g kg^−1^, respectively) and a combination with two essential oils known for their antimicrobial activity. We hypothesized that the selected microbial communities would react differently to the stress caused by maltodextrin-encapsulated essential oils. The serial dilution method assessed the number of colony-forming units (CFU) of bacteria, fungi, and actinomycetes. As the goal of microencapsulation was to prolong the effect of essential oils, their reaction was observed over a longer period. The soil microbial populations were examined in sandy and loamy soil at 1, 7, 14, and 78 days after encapsulated essential oils were mixed with the soil samples. In both types of soil, a significant increase in bacteria and actinomycetes was observed with maltodextrin in both doses. Encapsulated peppermint and caraway oils had different effects on microbes, both inhibitory and stimulatory. It is also important to note that peppermint with a smaller dose of maltodextrin significantly inhibited the growth of fungi in sandy soil in all measurements, as well as that caraway oil with a higher dose of maltodextrin significantly stimulated the growth of bacteria and actinomycetes in sandy soil. The higher dose of maltodextrin could explain this stimulation. Further research is recommended to test different doses of essential oils and maltodextrin, which would lead to the optimal dose of both wall and core materials.

## 1. Introduction

Essential oils contain a wide range of volatile molecules which possess several biological activities [1]. Due to their bactericidal, fungicidal, virucidal, and antioxidant properties, the application of essential oils in plant protection preparations has been known for a long time [2]. These non-synthetic preparations have gained popularity in organic agriculture as possible candidates for natural, botanical pesticides [3,4]. The most common problem faced by manufacturers of plant protection preparations based on essential oils is their stability, i.e., volatility. The control of their high volatility is the main challenge that has been solved by developing various techniques [5]. For this reason, in addition to essential oils, various wetting agents are added to the preparations, which prolong the effect of the active substance of the essential oil on the targeted plant organ.

Recently, microencapsulation of essential oils has begun to be used to achieve a delayed release and prolonged action. Encapsulation represents a viable and efficient approach to increase the physical stability of essential oils and protection from evaporation. Due to its narrow size range, it enables a controlled release and enhanced bioactivity [2]. The role of microencapsulation is to create a barrier that avoids chemical reactions and enables the controlled release of its core constituents at specific moments or over a prolonged period [6]. Selecting the right wall material for encapsulation in the case of essential oils can be very challenging. The wall material determines the core material’s encapsulation efficiency, stability, and level of protection. Materials mainly used in the microencapsulation of essential oils are different types of synthetic polymers and natural biomaterials (usually carbohydrates and proteins) [7]. The common material for the microcapsules carrier is maltodextrin, a carbohydrate containing D-glucose with its polymers and oligomers, with a glucose equivalent (DE) lower than 20. It is a product of acidic or enzymatic hydrolysis of starch [8]. Maltodextrins have functional, stabilizing, fluffing, filling, and freshness-extending properties [9,10]. As a carrier in microcapsules, they are characterized by several advantages, such as low price, good water solubility, availability in various variants of glucose equivalent (DE), formation of low-viscosity solutions, no off flavor, and the presence of glassy barrier coatings, which has a favorable effect on the stability of microcapsules. The main disadvantage of maltodextrins is their lack of emulsifying properties [9].

Both peppermint and caraway oil display several agro-biological activities [11,12] and are potential sources of botanical pesticides. Some authors accentuate soil application of these oils as microcapsules for effective pest and weed control [13,14,15,16]. Peppermint (*Mentha piperita* L.) is a perennial herb from the Lamiaceae family [17], originating from the Mediterranean region but cultivated all over the world [18]. The essential oil obtained from this plant has long been used in various forms, such as in managing plant pathogens and insect pests, traditional medicine, culinary, and cosmetics [19]. Peppermint oil with menthol and menthone as the main components exhibits high antimicrobial activities against Gram-positive and Gram-negative bacteria, yeast, and fungi [20]. These compounds have been successfully commercialized in the industry as antimicrobials/insecticidal agents [19]. Caraway (*Carum carvi* L.) belongs to the Apiaceae family and probably originates from Middle Asia. Caraway essential oil has antioxidant, insecticidal, antibacterial, fungicidal, acaricidal, molluscicidal, and larvicidal activities [21]. For example, S-(+)-carvone, the main compound of caraway oil, inhibits the growth of some bacteria and fungi and can be used as a natural sprout suppressant during potato storage [22]. Antimicrobial activity of the caraway oil against, e.g., protozoa, bacteria, mold fungi, and dermatophytes was also confirmed by [23,24].

Many reports mention the use of essential oils to protect above-ground plant organs and store raw materials and seeds, but only a few address the effects of treatments on soil microbiota. Previous research has shown that the soil’s microbial community reacts differently to various types of stress because their behavior depends on species mortality, species-specific resistance, the type of interactions among the members of the community, different microbial strains, and colonies, and, considering this, they should be evaluated at the community level [25,26]. Moreover, the efficiency of encapsulated essential oils in soil depends on the soil type, so a higher content of clay and loam could reduce the inhibitory effect of essential oils [27]. Research on this topic could be interesting both from the aspect of suppressing undesirable microorganisms in the soil and also as a study of the risk of undesirable effects of aerosol application on beneficial microorganisms in the surface layer of the soil.

This research aimed to study the effects of microencapsulated essential oils on sandy and loamy soil microbiota in vitro. For this purpose, two essential oils, very active in terms of their effect on them, were chosen, peppermint oil and caraway oil. Maltodextrin was also chosen as the universal carrier for microencapsulated essential oils. Dose and time effect dependencies were recorded and presented in this work.

## 2. Results

The number of colony-forming units (CFUs) of microorganisms in the soils studied in this experiment was in the lower ranges (Table 1). Depending on the type of soil, the way it is used, or the season, one gram of it can contain from 10^6^ to 10^10^ CFUs of culturable bacteria, from 10^5^ to 10^7^ of actinomycetes, and from 10^4^ to 10^8^ of fungi [28,29,30,31].

### 2.1. Effects on Bacteria

**Figure 1:**
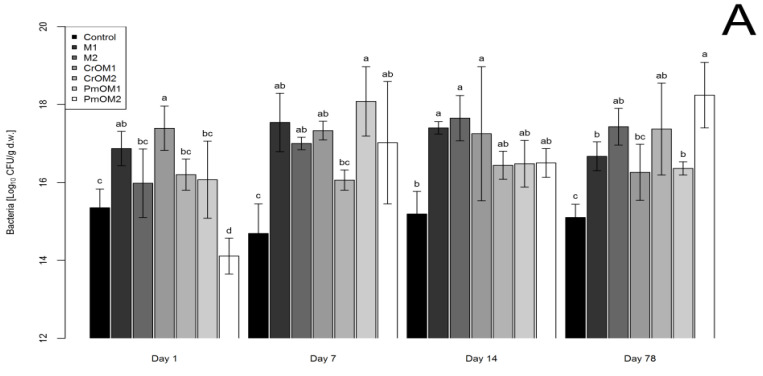

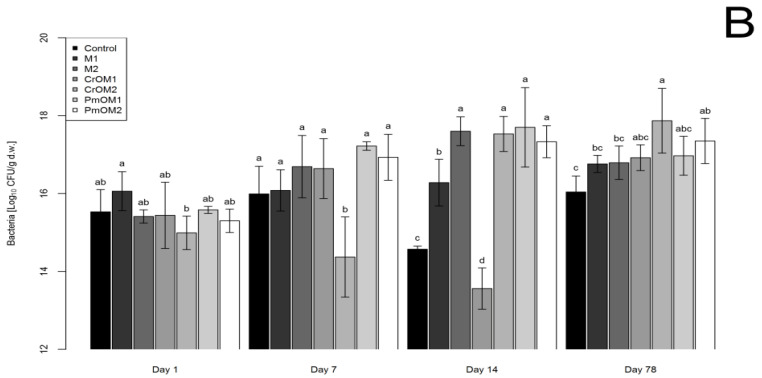
The abundance of bacteria in sandy (**A**) and loamy (**B**) soils depending on different treatments with maltodextrin (M1 and M2) and encapsulated essential oils of caraway (CrOM1 and CrOM2) and peppermint (PmOM1 and PmOM2). 1 and 2 refer to doses of microcapsules, 50 and 200 g per 1 kg of soil, respectively. Letters on error bars denote statistical significance among groups according to Duncan’s MLR post hoc test at level *p* < 0.05.

The effects of different treatments with microencapsulated essential oils on the soil’s microbiota in the case of sandy and loamy soil observed at different time intervals are shown in Figure 1A and Figure 1B, respectively and in Table S1. These logarithmically transformed data show a positive effect on the number of bacteria in the soil for almost all treatments against the control for sandy and loamy soils. In the case of sandy soil, only the encapsulated peppermint oil had antimicrobial activity, but only on the first day after mixing (DAM) the microencapsulated essential oils with soil. All treatments showed a positive effect in the remaining days compared to the control. At 7 DAM, all treatments, except microencapsulated caraway oil in a higher dose (CrOM2), were statistically different from the control. At 14 DAM, the maltodextrin treatments in both doses and CrOM1 (microencapsulated caraway oil in a lower dose) were statistically different from the control. The remaining treatments belonged to the same group as the control. In the measurements at 78 DAM, a significant increase in the number of bacteria was observed in all treatments compared to the control, except for CrOM1, which was not significantly different from the control.

In the case of loamy soil, at 1 DAM, there was no significant difference in the number of bacteria compared to the control in all treatments. At 7 DAM, only CrOM2 significantly reduced the number of bacteria compared to the control, while the other treatments were in the same group as the control. At 14 DAM, all treatments showed a significant stimulating effect on the growth of bacteria, except for CrOM1, which significantly inhibited their growth. In the measurements at 78 DAM, all treatments were in the same group as the control, except for CrOM2 and PmOM2 (microencapsulated peppermint oil in a higher dose), which significantly stimulated the growth of bacteria.

### 2.2. Effects on Fungi

**Figure 2:**
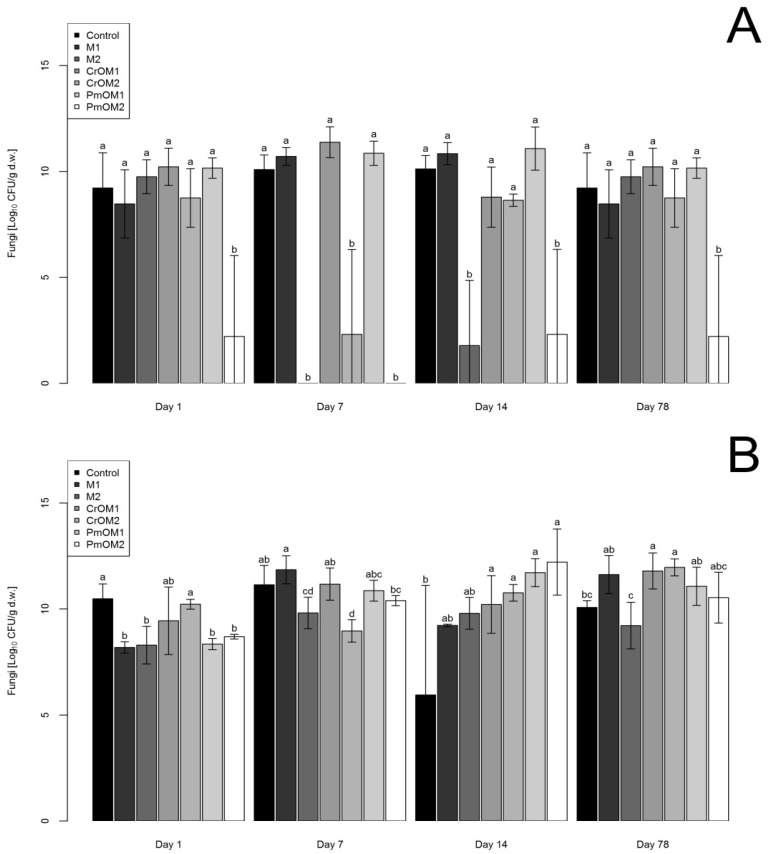
The abundance of fungi in sandy (**A**) and loamy (**B**) soils depending on different treatments with maltodextrin (M1 and M2) and encapsulated essential oils of caraway (CrOM1 and CrOM2) and peppermint (PmOM1 and PmOM2). 1 and 2 refer to doses of microcapsules, 50 and 200 g per 1 kg of soil, respectively. Letters on error bars denote statistical significance among groups according to Duncan’s MLR post hoc test at level *p* < 0.05.

The results shown in Figure 2A and Figure 2B, respectively and in Table S2, present the effects of different treatments on the growth of fungi in sandy and loamy soil, respectively. In the case of sandy soil, fungal abundance did not change compared to the control, but in some cases, an inhibitory effect was observed. In all measurement sections, PmOM2 affected the inhibition of fungal growth. At 1 DAM, only PmOM2 was statistically different from the control, showing a significant inhibitory effect on fungal growth. At 7 DAM, a low dose of maltodextrin in each combination (M2, CrOM2, and PmOM2) showed almost total inhibition of fungal growth, while the other treatments were the same as the control. In the measurements at 14 DAM, M2 and PmOM2 also inhibited fungal growth, while the other treatments had no effect compared to the control. At 78 DAM, the number of fungi was almost the same as on the first DAM, with the inhibitory effect of PmOM2 and the other treatments, which did not affect the growth of fungi compared to the control.

In the case of loamy soil, inhibition of fungal growth was observed at 1 and 7 DAM, while, in the remaining measurement days, most treatments had a stimulating effect on the growth of fungi. At 1 DAM, both doses of maltodextrin (M1 and M2) and peppermint oil (PmOM1 and PmOM2) showed inhibition of fungal growth, while other treatments had no effect compared to the control. Results for all treatments, at 7 DAM, were as for the control, except for M2 and CrOM2, which significantly reduced fungal growth. In the measurements at 14 DAM, fungal growth was observed in all treatments except for M1 and M2, which did not differ from the control. Measurements at 78 DAM showed that both doses of caraway oil and maltodextrin (CrOM1 and CrOM2) had a stimulating effect on fungal growth, while the other treatments had no significant effect.

### 2.3. Effects on Actinomycetes

**Figure 3:**
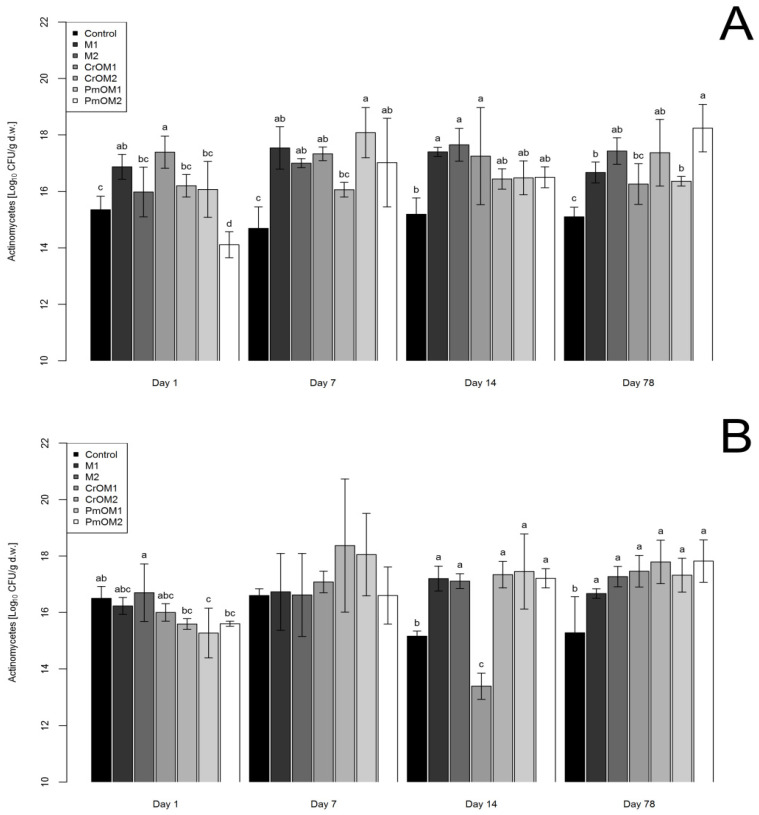
The abundance of actinomycetes in sandy (**A**) and loamy (**B**) soils depending on different treatments with maltodextrin (M1 and M2) and encapsulated essential oils of caraway (CrOM1 and CrOM2) and peppermint (PmOM1 and PmOM2). 1 and 2 refer to doses of microcapsules, 50 and 200 g per 1 kg of soil, respectively. Letters on error bars denote statistical significance among groups according to Duncan’s MLR post hoc test at level *p* < 0.05.

The effect of different treatments on the growth of actinomycetes in sandy and loamy soil is shown in Figure 3A and Figure 3B, respectively, and in Table S3. In the sandy and loamy soils, the treatments mainly stimulated the growth of actinomycetes. Only the encapsulated peppermint oil with a lower dose of maltodextrin had an antimicrobial activity at 1 DAM in sandy soil. Treatments M1 and CrOM1 influenced the increased growth of actinomycetes, while the other treatments presented the same as the control. At 7 DAM, in all treatments, the stimulation of growth of actinomycetes was observed, except for CrOM2, which was the same as the control. In the measurements at 14 DAM, both doses of maltodextrin (M1 and M2) and CrOM1 positively affected actinomycetes’ growth, while the other treatments had no significant effect compared to the control. At 78 DAM, all treatments stimulated the growth of actinomycetes, except for CrOM1, which was the same as the control.

In the case of loamy soil, at 1 and 7 DAM, there were no significant changes in the number of actinomycetes compared to the control, except for PmOM1, which inhibited their growth only at 1 DAM. A significant stimulating effect in all treatments was measured at 14 and 78 DAM, except for encapsulated caraway oil with a higher dose of maltodextrin (CrOM1), which significantly inhibited the growth of actinomycetes at 14 DAM.

The best models for the influence of the day after mixing the microencapsulated essential oils with the soils (DAM (*x*)) on CFUs of bacteria, fungi, and actinomycetes (*y*) are presented in the regression results in Table 2. From 14 DAM, the microcapsules with caraway oil in lower doses (CrOM2) and peppermint oil in both doses (PmOM1 and PmOM2) stimulated bacteria’s CFUs in both soils, as also confirmed by the high fit of the regression analyses, with a high value of determination coefficient R^2^, which shows a positive correlation between the application of microcapsules with peppermint oil and bacteria growth, especially for loamy soil. Regardless of high correlation coefficient values for the effect of DAM on fungi and both microcapsules, the best fit was found for microcapsules with caraway essential oil in a lower dose (CrOM1) and only for loamy soil (Table 2).

## 3. Discussion

The number of microorganisms varies in different soil types and conditions, with a predominance of bacteria [32]. The number of bacteria and actinomycetes in different soil types ranges from 10^6^ to 10^9^ g^−1^ in dry soil. Fungi are less numerous, usually around 10^4^ to 10^6^ g^−1^ in dry soil [32,33]. However, the number of microorganisms determined in the soil depends on many factors. The soil type, humidity, plant density, season of the year, or even the culture medium used in the laboratory could affect the microbial community profile. In the soil samples tested in this experiment, the overall number of different microorganisms, i.e., bacteria, actinomycetes, and fungi, was low but within the given range limits. A higher number of microorganisms in loamy soil than in sandy soil could result from richer soil nutrients.

The addition of maltodextrin, or as a wall material, in both doses mainly influenced the growth of bacteria and actinomycetes. This may be due to the fact that maltodextrin is built from D-glucose and its polymers and oligomers serve as C and energy sources for microorganisms [9,10], thus stimulating the transition of microorganisms from dormant or potentially active to active stages [34]. In the case of fungi, maltodextrin mainly did not affect their growth in all measurements in both soil types. In smaller doses, it inhibited fungal growth seven and 14 days after its addition in sandy soil and after one and seven days in loamy soil.

Encapsulated essential oils had different effects on soil microorganisms, both stimulating and inhibitory. Peppermint essential oil with a higher dose of maltodextrin mainly stimulated the growth of bacteria in both soil types. The encapsulation of peppermint oil with a lower dose of maltodextrin inhibits the growth of bacteria only on the first day in sandy soil. In later measurements, a stimulation effect was observed. Stimulating effects of *Mentha spicata* essential oil on the microbial community were reported earlier [26,35]; the latter observed a special favoring effect on Gram-positive bacteria that are more resistant to the denaturation of the cellular membranes. Stimulatory effects of essential oils on bacterial populations have also been repeatedly reported [36,37]. Furthermore, menthol, the main compound of the tested peppermint essential oil, showed low to intermediate antimicrobial activities against Gram-positive and Gram-negative bacteria [38].

In the case of fungi, encapsulated peppermint oil with a higher dose of maltodextrin did not affect their growth in sandy soil. They were significantly inhibited by peppermint oil with a lower dose of maltodextrin. This may also indicate a reduced inhibitory effect of the essential oil in the presence of a high dose of maltodextrin. Our results follow [19], which reported that different *Mentha* species, including *M. piperita,* possess a strong antifungal activity against tested plant fungi due to menthol being the main constituent. Antifungal effects of peppermint oil were also reported by other authors [20,26]. In the loamy soil, inhibition and stimulation were observed on the 1st and 7th day, respectively. Soković et al. [39] point out that the mycelial growth of the tested fungi reacted differently to the peppermint essential oil, which indicates that some fungi were able to overcome the effect of the essential oil or adapt to it. After its application in the soil, significant stimulation of soil fungi by *Mentha piperita* oil was observed after 28 days [35].

A different mode of action was observed in the case of actinomycetes. In sandy soil, peppermint oil stimulated the growth of actinomycetes on the 7th and 78th days, while in loamy soil, their number grew on the 14th and 78th days. The inhibitory effect was observed only on the 1st day of measurement. Previous researches indicate that *Mentha piperita* and *Mentha spicata* oils do not affect the growth of actinomycetes in the soil [26,35].

Caraway oil encapsulated with a higher dose of maltodextrin significantly increased the growth of bacteria in sandy soil. Its encapsulation with a lower dose of maltodextrin did not affect the growth of bacteria until the 78th day, when stimulation was also observed. Furthermore, the intermediate antimicrobial activity of caraway oil against some bacteria and fungi was reported by [22]. This might be due to carbohydrates from maltodextrin, which can stimulate intensive microbial growth and, consequently, accumulation of bacterial and especially fungal mucilage in the rhizosphere [40]. Inhibition of bacterial growth was observed only on the 7th and 14th days in loamy soil, depending on the dose of maltodextrin. A reduction in bacterial growth caused by encapsulated caraway oil with maltodextrin was also observed [14]. Iacobellis et al. [23] noticed a strong inhibitory effect of caraway oil against different bacterial genera responsible for plant diseases worldwide (*Clavibacter*, *Curtobacterium*, *Rhodococcus*, *Erwinia*, *Xanthomonas*, *Ralstonia*, and *Agrobacterium*).

Fungi reacted differently to treatment with encapsulated caraway oil. In sandy soil, encapsulated caraway oil did not affect fungi in any of the measurements. In both soil types, significant inhibition was only observed on the 7th day with encapsulation with a lower dose of maltodextrin. Poor antifungal activity of caraway oil in vitro and in vivo was also reported by [41], who examined its effect on postharvest diseases on tomato fruits. Contrarily, the inhibition of fungi with non-encapsulated caraway oil was observed by many authors [20,42,43]. Sokovic et al. [39] mentioned that limonene exhibits intermediate fungistatic and fungicidal activity, while carvone, the main compound in our essential oil, shows much higher antifungal activity. Strong carvone activity against fungi was also evidenced by [44].

The stimulation effect on actinomycetes was observed with caraway oil with a higher dose of maltodextrin. In comparison, a lower dose had an inhibitory effect, except on the 78th day when a stimulating effect was noted. In loamy soil, stimulation was mainly observed in later measurements.

The stimulatory effect of microencapsulated essential oils on the soil microbiota that was observed in the later measurements could result from the presence of maltodextrin coating and/or the rapid degradation of the essential oils by microbiota, which was reported by [16,45]. In summary, the microencapsulated peppermint and caraway oils display stimulatory or/and inhibitory effects on different groups of microorganisms in sandy and loamy soils. However, the overall result of microcapsules on microorganisms is a combined effect of the core oil and its maltodextrin coating. Maltodextrin exhibits a longer-lasting stimulatory effect on different groups of soil microbiota. Further research is recommended to test different doses of essential oils and maltodextrin for optimizing both the wall and the core materials.

## 4. Materials and Methods

The microcapsules with maltodextrin as a carrier and caraway or peppermint oil as a core were purchased from a commercial producer (Hoffmann Aroma, Zamysłowo, Poland). The oil content and chemical composition were characterized and presented in our previous work [13,15] and are summarized in Table 3.

Topsoil layers (0–25 cm) from arable fields in Mydlniki, south of Poland, were collected in the spring of 2019. One of the soils was sandy, and the other was loamy. The soils were kept in the laboratory at room temperature until the experiments. 

The air-dry soils were sieved through a 2 mm sieve to remove all the impurities. The basic physicochemical properties of the soils were estimated (Table 4). The following methods were applied: soil pH in 1 mol ∙ dm^−3^ KCl (soil:solution = 1:2.5) was determined potentiometrically [46], whereas we estimated organic carbon using the Tiurin method. The content of Ca, Mg, K, and *p* in soil samples was determined after mineralization in the muffle furnace and the dissolution of the ash solution in a mixture of HNO_3_ and HClO_4_ (3:1 *v*/*v*) [47]. The element contents in the solutions were determined using an inductively coupled plasma atomic emission spectrometer (ICP-AES) by Perkin-Elmer, model Optima 7300 DV. Each sample was subjected to chemical analysis twice. The quality of determinations was verified based on the element determinations obtained for certified materials: CRM 16-050 (total element content).

50 cm^3^ of soil was measured into sterile polystyrene opaque containers. Appropriate doses of microcapsules (0.18 g and 0.72 g) with peppermint oil, caraway oil, or maltodextrin alone were weighed, added to separate containers with soil, and mixed. The soil mixtures with microcapsules corresponded to doses of 50 and 200 g of microencapsulated oils or maltodextrin carrier per 1 kg of soil. Containers with soils were only used as a control. The dry matter of the soil was determined according to Polish standards (PN-ISO 11465:1999) [48].

For the microbiological soil analysis, the serial dilution method was used [49]. Dilutions from 10^−1^ to 10^−6^ were prepared, bacteria were cultivated on Tripticasein Soy Lab-Agar (Biomaxima), fungi on Malt Extract Agar (Biomaxima), and actinobacteria on Actinomycetes Cultivation Medium [50]. The cultivation of the bacteria was carried out for 24 h at 37 °C and next for 72 h at 22 °C, fungi for 5 days at 26 °C, and actinobacteria for 7 days at 26 °C. The grown colonies were counted, and the results were recalculated and expressed as the number of CFUs (Colony Forming Units) per 1 g of dry soil (CFU/g DW of soil) [51].

The microbiological analyses were performed on the first, seventh, fourtheen, and seventy-eighth day after mixing with soil, in triplicate. Between the analyses, the soil samples with the microcapsules were stored in the dark at about 22 °C.

### Statistical Analysis

All counting measurements were done in triplicate. Since soil microbiota showed exponential growth, data were logarithmically transformed with the formula log_10_(x + 1) before performing a one-way ANOVA. Results are presented graphically in bar plots with statistical differences among means denoted with letters on error bars according to Duncan’s MLR post hoc test at *p* < 0.05. All statistical analyses were performed utilizing R-CRAN Statistical Procedures for Agricultural Research, package Agricolae.

The relationships between the observed traits were estimated using Pearson’s linear correlation coefficients. The influence of time (days) on values of observed traits (bacteria, fungi, and actinomycetes) was assessed by regression analysis [52]. The GenStat v. 18 statistical software package (VSN International) was used for these analyses.

## Figures and Tables

**Table 1:** The mean number of colony-forming units (CFU). Results are expressed in CFU/g dry weight of soil.

Group of Microorganisms	Sandy Soil	Loamy Soil
	[CFU/g DW of Soil]	[CFU/g DW of Soil]
Bacteria	5.6 × 10^6^	7 × 10^6^
Actinomycetes	4 × 10^6^	10 × 10^6^
Fungi	2.7 × 10^4^	3.9 × 10^4^

**Table 2:** The best models for the influence of time (days) after mixing soils with the microencapsulated essential oils (x) on the soil populations of bacteria, fungi, and actinomycetes (y) resulted from the regression analysis.

		Bacteria		Fungi		Actinomycetes	
Additive	Soil	Model	R^2^ [in %]	Model	R^2^ [in %]	Model	R^2^ [in %]
Control	Loamy	y = 0.0051x^2^ − 0.3855x + 8.6848	16.7	y = −0.0043x^2^ + 0.2546x + 30.27	0.75	y = 0.0114x^2^ − 1.0501x + 18.686	52.13
Control	Sandy	y = −0.0035x^2^ + 0.3265x + 3.0204	48.27	y = 37.558x^−0.258^	19.6	y = −0.233ln(x) + 4.5192	4.44
M1	Loamy	y = −0.0018x^2^ + 0.2652x + 9.4873	39.2	y = −0.3391x^2^ + 28.238x + 1.0399	43.6	y = −0.0235x^2^ + 1.8749x + 13.908	14.15
M1	Sandy	y = −0.023x^2^ + 1.9201x + 2.1614	45.62	y = −0.0544x^2^ + 4.228x + 11.776	57.49	y = −0.019x^2^ + 1.359x + 27.509	28.64
M2	Loamy	y = −0.0456x^2^ + 3.8267x − 0.2736	69.76	y = 6.0015x^0.1939^	12.72	y = 16.225x^0.1436^	7.88
M2	Sandy	y = −0.0581x^2^ + 5.1497x − 6.3094	50.13	y = 0.0421x^2^ − 3.4406x + 33.904	42.77	y = −0.0418x^2^ + 3.7111x + 4.9978	58.42
CrOM1	Loamy	y = 0.0087x^2^ − 0.5434x + 12.351	33.26	y = −0.0096x^2^ + 2.3335x + 35.512	48.47	y = 0.015x^2^ − 0.8572x + 17.551	50.44
CrOM1	Sandy	y = 8.4257x^0.1483^	7.28	y = 0.0145x^2^ − 1.3838x + 55.557	2.65	y = 44.896x^−0.25^	17.79
CrOM2	Loamy	y = 2.0117x^0.75^	50.81	y = 0.0363x^2^ − 1.0267x + 23.899	86.11	y = 11.176x^0.4669^	27.74
CrOM2	Sandy	y = −0.0328x^2^ + 2.8279x − 5.8971	50.64	y = 0.002x^2^ − 0.0903x + 4.7247	24.94	y = 0.0057x^2^ + 0.1382x + 10.328	34.8
PmOM1	Loamy	y = −0.0647x^2^ + 5.3902x − 1.1058	56.93	y = 6.5295x^0.598^	52.54	y = 8.9653x^0.4534^	25.79
PmOM1	Sandy	y = −0.0117x^2^ + 0.8046x + 15.638	11.44	y = −0.093x^2^ + 7.5897x + 1.5533	50.91	y = −0.0097x^2^	8.53
PmOM2	Loamy	y = 6.2719x^0.4813^	65.66	y = −0.1445x^2^ + 12.252x − 19.2	57.06	y = 6.2305x^0.5203^	69.06
PmOM2	Sandy	y = 2.2999x^0.9165^	85.53	y = 0.0028x^2^ − 0.253x + 2.7545	58.62	y = 1.8474x^0.9122^	71.4

**Table 3:** Content and main compounds of peppermint and caraway essential oils in the microcapsules with maltodextrin as a carrier.

Characteristics	Peppermint Oil	Caraway oil
Content of oil (*v*/*w* %)	9.6	6.5
(−)-Menthone (%)	20.6	--
(−)-Menthol (%)	60.1	--
D-Limonene (%)	--	15.2
S-(+)-Carvone (%)	--	79.9

**Table 4:** Chemical characteristics of top soils used in the experiments.

Characteristics	Sandy Soil	Loamy Soil
pH _KCl_	6.8	6.5
Ca (g kg^−1^)	0.94	2.45
K (g kg^−1^)	0.49	1.65
P (g kg^−1^)	0.22	0.45
Mg (g kg^−1^)	0.36	1.68
C organic (g kg^−1^)	13.25	20.11
Soil dry matter (%)	87.9	87.5

## Data Availability

The data presented in this study are available on request from the corresponding author.

## Supplementary Materials

The following supporting information can be downloaded at: https://www.mdpi.com/article/10.3390/plants11233343/s1, Table S1: Mean values [in millions], standard deviations and Fisher’s least significant differences (LSDs) for bacteria; Table S2: Mean values [in thousands], standard deviations and Fisher’s least significant differences (LSDs) for fungi; Table S3: Mean values [in millions], standard deviations and Fisher’s least significant differences (LSDs) for actinomycetes.
