# Supplementary material for: Maltodextrin-Coated Peppermint and Caraway Essential Oils Effects on Soil Microbiota
Source: Plants (Basel). 2022 Dec 2;11(23):3343. doi: 10.3390/plants11233343 (PMC9739318; doi:10.3390/plants11233343)
Supplement: Supplementary file 1 [file plants-11-03343-s001.zip › plants-1823328-supplementary.pdf]

Table S1. Mean values [in millions], standard deviations and Fisher's least significant differences (LSDs) for bacteria

Soil		Loamy soil		Sandy soil		Average	
Day	Microcapsule	Mean	s.d.	Mean	s.d.	Mean	s.d.
1	Control	6.22	3.82	3.86	2.12	5.04	3.05
	M1	10.13	4.29	2.18	1.02	6.16	5.17
	M2	4.98	0.83	5.54	2.11	5.26	1.47
	CrOM1	6.38	4.83	13.48	11.7	9.93	8.9
	CrOM2	3.46	1.6	1.9	1.49	2.68	1.63
	PmOM1	5.84	0.54	4.09	4.43	4.96	2.98
	PmOM2	4.58	1.48	2.17	1.05	3.37	1.75
7	Control	10.43	7.62	4.1	1.03	7.27	5.97
	M1	10.49	4.57	18.25	15.54	14.37	11.09
	M2	21.31	12.96	13.29	8.32	17.3	10.69
	CrOM1	19.93	11.58	18.11	13.54	19.02	11.31
	CrOM2	2.29	1.53	2.23	1.48	2.26	1.35
	PmOM1	30.2	3.43	45.56	21.54	37.88	16.16
	PmOM2	24.88	12.53	14.62	5.27	19.75	10.27
14	Control	2.14	0.18	7.44	0.66	4.79	2.93
	M1	13.24	7.96	22.59	8.65	17.91	9.03
	M2	45.88	17.34	61.36	34.67	53.62	25.94
	CrOM1	0.84	0.36	13.46	3.47	7.15	7.26
	CrOM2	43.81	17.69	32.42	19.08	38.11	17.6
	PmOM1	63.34	42.01	11.88	7.05	37.61	38.99
	PmOM2	35.58	15.49	41.14	21.67	38.36	17.12
78	Control	9.75	3.73	7.29	2.74	8.52	3.22
	M1	19.24	4.23	11.78	5.82	15.51	6.12
	M2	20.77	7.65	41.66	29.47	31.21	22.4
	CrOM1	23.01	6.81	14.93	1.08	18.97	6.22
	CrOM2	73.63	65.69	15.09	9.3	44.36	52.81
	PmOM1	25.4	11.06	7.47	1.02	16.44	12.07
	PmOM2	37.78	17.46	127.75	83.95	82.77	73.27
	Control	7.13	5.25	5.67	2.36		
	M1	13.28	6.04	13.7	11.33		
	M2	23.24	18.15	30.46	30.57		
	CrOM1	12.54	11.38	14.99	8.03		
	CrOM2	30.8	42.6	12.91	15.88		
	PmOM1	31.19	28.49	17.25	19.92		
	PmOM2	25.7	17.78	46.42	63.19		
	Day 1	5.94	3.2	4.74	5.65		
	Day 7	17.08	11.83	16.59	16.8		
	Day 14	29.26	28.13	27.18	23.47		
	Day 78	29.94	29.75	32.28	50.24		
$LSD_{0.05}$		Day (D): 8.08; Microcapsule (M): 10.69; Soil (S): 5.71; DM: 21.38; DS: 11.43; MS: 15.11; DMS: 30.23					

Table S2. Mean values [in thousands], standard deviations and Fisher's least significant differences (LSDs) for fungi

Soil		Loamy soil		Sandy soil		Average	
Day	Microcapsule	Mean	s.d.	Mean	s.d.	Mean	s.d.
1	Control	3.65	0.9	34.31	11.99	18.98	18.43
	M1	40.68	21.45	11.74	7.69	26.21	21.42
	M2	4.91	3.06	36.41	28.42	20.66	24.99
	CrOM1	21.81	17.15	26.75	4.71	24.28	11.57
	CrOM2	27.89	6.02	6.55	3.91	17.22	12.54
	PmOM1	4.27	1.07	5.15	4.49	4.71	2.96
	PmOM2	5.94	0.61	3.06	1.08	4.5	1.76
7	Control	85.95	54.35	28.38	20.17	57.17	48.35
	M1	158.45	84.16	47.19	17.48	102.82	81.67
	M2	22.08	17.37	0	0	11.04	16.34
	CrOM1	83.65	47.17	101.84	56.18	92.75	47.46
	CrOM2	8.47	3.67	0.34	0.59	4.4	5.03
	PmOM1	56.12	23.38	57.98	31.9	57.05	25.03
	PmOM2	33.24	8.08	0	0	16.62	18.91
14	Control	5.29	5.29	27.97	14.52	16.63	15.81
	M1	342.01	267.76	55.96	31.48	198.99	231.56
	M2	10.16	0.61	0.07	0.12	5.11	5.54
	CrOM1	49.77	20.67	10.72	9.62	30.24	25.79
	CrOM2	21.78	17.68	5.82	1.57	13.8	14.23
	PmOM1	44.92	45.77	85.56	61.87	65.24	53.52
	PmOM2	137.44	70.08	0.34	0.59	68.89	87.2
78	Control	24.46	8.02	18.28	15.49	21.37	11.54
	M1	140.08	98.56	10.67	14.57	75.37	94.84
	M2	15.22	16.93	21.51	18.63	18.36	16.29
	CrOM1	159.2	95.18	36.06	33.75	97.63	92.88
	CrOM2	164.86	57.11	9.72	7.31	87.29	92.44
	PmOM1	81.49	59.59	27.9	14.16	54.69	48.6
	PmOM2	56.93	56.27	0.25	0.43	28.59	47.23
	Control	29.84	42.09	27.24	14.77		
	M1	170.3	170.54	31.39	27.21		
	M2	13.09	12.35	14.5	21.64		
	CrOM1	78.61	71.19	43.84	45.99		
	CrOM2	55.75	71	5.61	5.04		
	PmOM1	46.7	44.41	44.15	43.91		
	PmOM2	58.38	64.1	0.91	1.42		
	Day 1	15.59	16.58	17.71	17.11		
	Day 7	63.99	60.54	33.68	42.58		
	Day 14	87.34	145.2	26.64	38.41		
	Day 78	91.75	80.62	17.77	18.71		
$LSD_{0.05}$		Day (D): 21.29; Microcapsule (M): 28.17; Soil (S): 15.06; DM: 56.34; DS: 30.11; MS: 39.84; DMS: 79.68					

Table S3. Mean values [in millions], standard deviations and Fisher's least significant differences (LSDs) for actinomycetes

Soil		Loamy soil		Sandy soil		Average	
Day	Microcapsule	Mean	s.d.	Mean	s.d.	Mean	s.d.
1	Control	15.39	5.54	5.02	2.2	10.2	6.82
	M1	11.59	3.64	22.67	9.85	17.13	9
	M2	24.77	23.37	10.92	8	17.85	17.37
	CrOM1	9.16	2.66	39.78	22	24.47	21.86
	CrOM2	6	1.22	11.44	4.65	8.72	4.26
	PmOM1	5.59	5.08	12.81	10.88	9.2	8.56
	PmOM2	5.97	0.54	1.44	0.71	3.71	2.54
7	Control	16.45	4.14	2.84	1.71	9.64	7.97
	M1	34.28	44	48.53	27.22	41.4	33.64
	M2	27.55	24.19	24.38	4.01	25.97	15.6
	CrOM1	27.48	9.55	34.36	8.57	30.92	8.95
	CrOM2	355.92	518.89	9.63	2.54	182.77	379.05
	PmOM1	111.9	85.85	88.52	56.12	100.21	66.12
	PmOM2	21.48	16.19	55.37	77.23	38.43	53.25
14	Control	3.9	0.69	4.37	2.1	4.13	1.42
	M1	31.26	11.8	36.45	5.74	33.85	8.77
	M2	27.42	6.72	51.08	23.95	39.25	20.38
	CrOM1	0.7	0.32	83.07	123.86	41.89	90.4
	CrOM2	36.27	14.54	14.38	4.71	25.32	15.4
	PmOM1	57.19	42.58	15.92	7.77	36.56	35.5
	PmOM2	30.77	9.26	15.33	5.76	23.05	10.91
78	Control	6.45	5.1	3.77	1.37	5.11	3.65
	M1	17.47	3.04	18.1	6.63	17.79	4.62
	M2	32.9	10.92	39.83	16	36.36	12.82
	CrOM1	41.88	19.35	13.57	8.59	27.72	20.49
	CrOM2	62.64	36.73	55.77	63.49	59.21	46.54
	PmOM1	36.75	17.74	12.83	2.2	24.79	17.3
	PmOM2	65.05	41.7	101.14	60.59	83.1	50.55
	Control	10.55	6.79	4	1.8		
	M1	23.65	21.86	31.44	17.95		
	M2	28.16	15.65	31.55	20.45		
	CrOM1	19.81	19.11	42.69	60.01		
	CrOM2	115.21	265.98	22.8	33.77		
	PmOM1	52.86	58.03	32.52	41.81		
	PmOM2	30.82	29.85	43.32	58.32		
	Day 1	11.21	10.31	14.87	15.09		
	Day 7	85.01	204.34	37.66	42.14		
	Day 14	26.79	23.83	31.51	47.93		
	Day 78	37.59	28.51	35	43.18		
$LSD_{0.05}$		Day (D): 32.75; Microcapsule (M): 43.32; Soil (S): 23.16; DM: 86.64; DS: 46.31; MS: 61.27; DMS: 122.53					
